# Resurrection of the Butterfly-winged Comber, *Serranus papilionaceus* Valenciennes, 1832 (Teleostei, Serranidae) and its phylogenetic position within genus *Serranus*

**DOI:** 10.3897/zookeys.1017.60637

**Published:** 2021-02-12

**Authors:** Adriana Vella, Noel Vella, Carolina Acosta-Díaz

**Affiliations:** 1 Conservation Biology Research Group, Department of Biology, University of Malta, Msida, MSD2080, Malta Biological Conservation Research Foundation Hamrun Malta; 2 Biological Conservation Research Foundation, PO BOX 30, Hamrun, HMR 1000, Malta University of Malta Msida Malta

**Keywords:** Butterfly-winged Comber, Canary Islands, Mediterranean Sea, Painted Comber, phylogeny, taxonomy

## Abstract

The family Serranidae is represented by 92 genera and 579 valid species, with the genus *Serranus* Cuvier, 1816, containing 30 species. In this study, specimens of Butterfly-winged Comber, *Serranus papilionaceus* Valenciennes, 1832, were collected from the Canary Islands and compared morphologically and genetically to Painted Comber, *Serranus scriba* (Linnaeus, 1758), from the Mediterranean Sea. Morphological differences, especially in the colour banding pattern, were corroborated by genetic differences in mitochondrial (COI and ND2) and nuclear (Rhod and PTR) markers. The mitochondrial DNA markers revealed a high level of divergence and no shared haplotypes between the two species (interspecific divergence: COI 4.31%; ND2 8.68%), and a phylogenetic analysis showed that these two species are closely related sister species sharing common ancestry. This study is therefore offering to resurrect *S. papilionaceus* Valenciennes, 1832 as a valid species increasing the number of eastern Atlantic *Serranus* species to 11. This should direct new species-specific research, including its population conservation status assessment across its distribution.

## Introduction

The family Serranidae, which is composed of sea basses and groupers, has three subfamilies Anthiadinae, Epinepheline, and Serraninae, which are represented by 579 valid species in 72 genera (Parenti and Randall 2020). The genus *Serranus* (Perciformes, Serranidae) has 30 small reef-associated species (Iwamoto and Wirtz 2018; Horton et al. 2020), with the most recent additions to the genus *Serranus* being *S. aliceae* Carvalho Filho & Ferreira, 2013 from the Southwest Atlantic Ocean and *S. pulcher* Wirtz & Iwamoto, 2016, *S. drewesi* Iwamoto, 2018, and *S. inexpectatus* Wirtz & Iwamoto, 2018 from the Eastern Central Atlantic Ocean (Carvalho Filho and Ferreira 2013; Wirtz and Iwamoto 2016; Iwamoto and Wirtz 2018).

**Figure 1.**
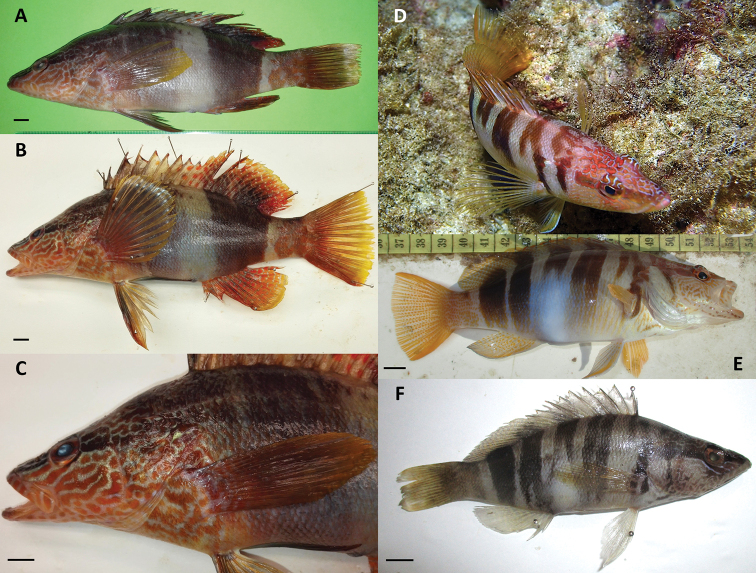
**A–C***Serranus papilionaceus***D–F***Serranus scriba*. Photos include details of specimens representing both species (underwater photography by Mr. P. Schembri, BICREF NGO). Scale bars: 1 cm (**A–C, E, F**).

Most of the species belonging to this genus are recorded in the coastal regions of the Atlantic Ocean, with 14 species occurring in the Western Atlantic and 10 species within the Eastern Atlantic. In addition to these, another six species inhabit the Eastern Pacific Ocean while another two occur in the Indian Ocean (Iwamoto and Wirtz 2018). Both the Mediterranean Sea and the Canary Islands are known to host the same four *Serranus* species (Dooley et al. 1985; Bauchot 1987): the Blacktail Comber, *S. atricauda* Günther, 1874, which in the Mediterranean is restricted to the western basin; the Comber, *S. cabrilla* (Linnaeus, 1758); the Brown Comber, *S. hepatus* (Linnaeus, 1758); and the Painted Comber, *S. scriba* (Linnaeus, 1758). However, analysing photos of specimens commonly identified as *S. scriba* collected off the Canary Islands, we noticed distinct differences in colour pattern when compared to the specimens from the Mediterranean Sea, including Malta (Fig. 1), as was already observed by Iwamoto and Wirtz (2018). These notable differences led us to further investigate the species’ identity using morphological and genetic analyses, while reviewing first descriptions and museum specimens to accurately identify the species in question.

## Materials and methods

### Morphological analyses of sampled specimens

**Figure 2.**
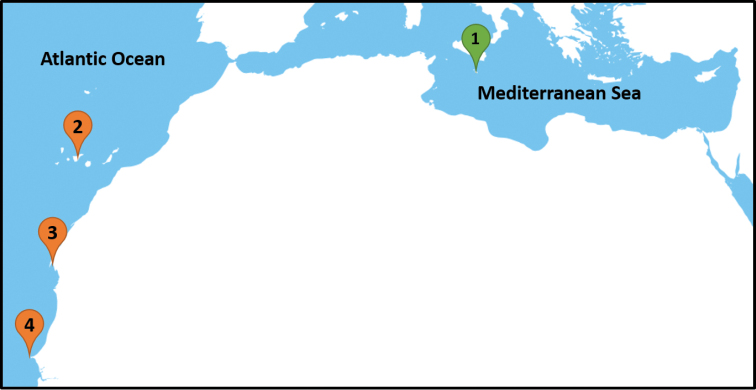
A map showing: the sampling locations for this study [1 – Malta, 2 – Canary Islands]; and the locations from where *Serranus papilionaceus* was recorded [2 – Canary Islands (Barker-Webb and Berthelot 1836; current study); 3 – Mauritania (MNHN-IC-1999-1053); 4 – Gorée, Senegal (Valenciennes, 1832; MNHN-IC-0000-7279; MNHN-IC-1896-0389)].

Specimens were collected by local coastal fishermen during regular fishing activities in the Canary Islands using traps for fish (*n* = 12) and Malta during shoreline fishing (*n* = 15) between 2016 and 2017 (Figs 1, 2; Suppl. material 1: Table S1). The total length of each specimen was measured to the nearest millimeter using electronic callipers, and tissue samples were taken from each specimen. All the specimens collected from Malta and five specimens collected from the Canary Islands were morphologically examined following Heemstra and Randall (1993) and Zorica et al. (2010), where various body parts were measured to the nearest millimeter using electronic callipers and counts of the soft and hard rays in the dorsal, pectoral, pelvic, anal, and caudal fins were counted.

### DNA and phylogenetic analyses

Total genomic DNA was extracted using GF-1 DNA Extraction Kit (Vivantis Technologies, Malaysia) and used as a template for amplifying two mitochondrial DNA (mtDNA) genes [cytochrome oxidase c subunit 1 gene (COI); and NADH dehydrogenase subunit 2 gene (ND2)], and two nuclear DNA (nDNA) genes [rhodopsin gene (Rhod); and si:ch211-105n9.1-like protein, hypothetical protein LOC564097 (PTR)]. The amplification protocols were carried out following literature in Table 1. The PCR products were sequenced using their respective forward and reverse primers through 3730XL Genetic Analyzer (Applied Biosystems, USA).

**Table 1.** A list of the primers and amplification protocols used to amplify the genes used in this study.

Gene	Primers	(5' to 3')	Reference
**COI**	FishF1	TCAACCAACCACAAAGACATTGGCAC	Ward et al. (2005)
	FishR1	TAGACTTCTGGGTGGCCAAAGAATCA	
**ND2**	ND2-MetF	AAGCTYTTGGGCCCATACC	Vella et al. (2017)
	ND2-TrpR	AGCTTTGAAGGCTTTTGGTYT	
**Rhod**	193F	CNTATGAATAYCCTCAGTACTACC	Chen et al. (2003)
	1039R	TGCTTGTTCATGCAGATGTAGA	
**PTR**	Ptr_F458	AGAATGGATWACCAACACYTACG	Chenhong et al. (2007)
	Ptr_R1248	TAAGGCACAGGATTGAGATGCT	

Sequences were trimmed and the complimentary sequences of each individual were assembled using Geneious R10 (Kearse et al. 2012). Sequences were manually checked for consistencies. The final sequences were deposited in GenBank under accession numbers: MW439283–MW439309 (COI); MW447416–MW447496 (ND2; Rhodo; PTR) (Suppl. material 1: Table S2)

The mtDNA haplotype diversity and nucleotide diversity indices for both species were calculated via Arlequin v. 3 (Excoffier and Lischer 2010), while the intraspecific and interspecific *p*-distance was measured using DnaSP (Rozas et al. 2017). Parsimony haplotype networks were constructed via TCS (Clement et al. 2000) to analyse the association between the various haplotypes identified during this study. For nDNA genes, Geneious R10 (Kearse et al. 2012) was used to identify single nucleotide polymorphism between the two species.

To evaluate the phylogenetic relationship of the resurrected taxon to that of other *Serranus* species, COI sequences were imported from BOLD (Ratnasingham and Hebert 2007) and GenBank (NCBI 2020). Further details of these sequences are included in Suppl. material 1: Table S3. All the COI sequences were aligned using ClustalW (Thompson et al. 1994) and the sequences were trimmed to 578 bp representing the smallest homologous sequence for this data set. The phylogenetic relationships between different *Serranus* species were evaluated through the construction of a phylogenetic tree using neighbour-joining analysis and the *p*-distance model (Collins et al. 2012; Srivathsan and Meier 2012; Collins and Cruickshank 2013). This analysis was conducted via MEGA v. 7 (Kumar et al. 2016) using 1000 bootstraps.

### Historical material

The following museum specimens’ photographs and describing manuscript diagrams were analysed carefully, comparing the historic image evidence, when available, with the photographs taken from specimens sampled for this study.

Descriptions of *S. scriba* and its possible synonyms were reviewed given the close resemblance between *S. scriba* and the species being resurrected. Relevant scientific descriptions evaluated for this purpose included: *Perca scriba* described by Linnaeus (1758: 292) and Linnaeus (1764: 86); *Perca marina* described by Brünnich (1768: 63); *Holocentrus fasciatus* described by Bloch (1790: 86); *Holocentrus marocannus* described by Bloch and Schneider (1801: 320); *Holocentrus argus* described by Spinola (1807: 372); *Serranus scriba* described by Cuvier and Valenciennes (1828: 214); and *Serranus papilionaceus* described by Valenciennes (1832: 471).

#### Holotypes

*Serranus scriba* originally described as *Perca scriba*Linnaeus 1758; NRM 442; holotype status follows Fernholm and Wheller (1983) and Parenti and Randall (2020) • *Holocentrus marocannus*Bloch and Schneider 1801; Morocco; ZMB5531 (left skin); a junior synonym to *Serranus scriba* as noted in Cuvier and Valenciennes (1828), Gunther (1859) and Parenti and Randall (2020).

#### Syntype

*Serranus papilionaceus*Valenciennes 1832; Gorée, Senegal, Atlantic Ocean; two specimens MNHN-IC-0000-7279.

#### Other material

*Serranus scriba*; Algeria; two specimens MNHN-IC-0000-7129 • *Serranus papilionaceus* (labelled as *Serranus scriba*); Dakar, Senegal; collected in 1896; MNHN-IC-1896-0389 • *Serranus papilionaceus* (labelled as *Serranus scriba*); Cap Blanc, Baie du Levrier, Mauritania; four specimens MNHN-IC-1999-1053.

## Results

Based on the currently used literature and morphological keys (Bauchot 1987; Lloris and Rucabado 1998; Iwamoto and Wirtz 2018), all specimens collected and analysed during this study (Suppl. material 1: Table S1) fit the body shape description of *S. scribasensu lato*; however, genetic data revealed divergence between the two forms. Based on the type specimens and descriptions evaluated in this study, we assigned the specimens collected from Malta to *S. scriba* in its traditional usage based on Cuvier and Valenciennes (1828). These specimens matched the museum specimens NRM 442 and MNHN-IC-0000-7129 (Suppl. material 1: Figs S1a, S2b). We assign the specimens collected from the Canary Islands to the resurrected taxon *S. papilionaceus*, as described by Valenciennes (1832) and matching the syntype specimens MNHN-IC-0000-7279 (Suppl. material 1: Fig. S2a) and museum specimens MNHN-IC-1896-0389 and MNHN-IC-1999-1053.

### Morphology

#### Meristic counts and measures

Meristic counts (Table 2) indicated a general overlap between the counts of the two species investigated, with only the anal soft fin rays counts showing a difference (7 in *S. scriba* and 8 in *S. papilionaceus*). Though the meristic measures (Table 3) overlapped between the two species, the specimens investigated also showed a longer average dorsal fin length in *S. papilionaceus* than in *S. scriba* (46.3% TL ± 1.1 and 41.4% TL ± 1.4, respectively). Extended morphometric studies on *S. papilionaceus* covering a wider distribution and life-stages are required to corroborate the significance of these differences. Consequently, the most important identifying morphological characteristic feature between the two species would be their body colouration patterns described below.

**Table 2.** Meristic counts for *Serranus scriba*, its synonyms^1^ (Parenti and Randall 2020) and *Serranus papilionaceus*. Values in brackets represent the mean and SD for counts that have a range. ^2^ Questionable locality (Fernholm and Wheller 1983).

Species name	References (sample size)	Meristic counts					Locality
		Dorsal	Pectoral	Ventral	Anal	Caudal	
*** Perca scriba***	Linnaeus, 1758	10 / 15	13	1 / 5	3 / 7	15	America (Linnaeus, 1764)^2^
* Perca marina* ^1^	Brünnich, 1768	10 / 16	13	1 / 5	3 / 8	15	Mediterranean
* Holocentrus fasciatus* ^1^	Bloch, 1790	10 / 15	13	1 / 5	3 / 7	16	
* Holocentrus marocannus* ^1^	Bloch & Schneider, 1801	10 / 16	15	1 / 5	3 / 7	18	Morocco
* Serranus scriba*	Cuvier & Valenciennes, 1828	10 / 14	13	1 / 5	3 / 7	17	Mediterranean: France, Malta, Italy and Egypt
* Serranus scriba*	Zorica et al. 2010	10 / 14–17	12–16	1 / 4–6	3 / 7–8	15–18	Trogir, Turkey
	(n = 253)	(15.01 ±2.04)	(14.10 ±1.15)	(4.97 ±0.49)	(7.03 ±0.82)	(16.59 ±1.36)	
* Serranus scriba*	current work	10 / 15	13–14	1 / 5	3 / 7	15–17	Malta
	(n = 15)		(13.27 ±0.46)			(16.67 ±0.62)	
* Serranus papilionaceus*	Valenciennes, 1832	10 / 15	16	1 / 5	3 / 8	17	Gorée, Senegal
* Serranus papilionaceus*	current work	10 / 15–16	14–15	1 / 5	3 / 8	17	Canary Islands
	(n = 5)	(15.40 ±0.55)	(14.60 ±0.55)				

#### Colour patterns

The *S. scriba* (Fig. 1) specimens analysed in this study had nine dorso-ventral brown bands. The first band was observed to originate dorsally over the head and fade ventrally just behind the preopercular area. The second band, starting in front of the origin of the dorsal fin, was found to touch the back of the operculum and end at the origin of the pectoral fin. The third, shorter band originating at the first hard spine of the dorsal fin, fades away ventrally. The fourth and fifth band originating at the membrane between the fourth and the seventh hard spine of the dorsal fin and fade away ventrally. They are usually paired up and at times are nearly overlapping. The sixth and seventh band originating at the membrane between the eighth hard spine and the first soft spine of the dorsal fin, may at times be fused, and fade away ventrally into a bluish-violet blotch located over the abdomen area. The eighth and ninth bands are much wider than the former bands, appearing as two fused bands, especially when they fork out ventrally. These two wider bands originate from the soft spines of the dorsal fin and fade away ventrally as they approach the anal fin. A brown banding pattern on the tail peduncle is not always present. The pectoral fins, pelvic fins, anal fin, and tail peduncle together with the tail, are brownish-orange. A faint bluish colouration on the outer side of the pelvic fins and anal fin membrane is present. The pelvic fins, anal fin, dorsal fin, tail peduncle, and tail have red-orange spots, while at the tip of each hard spine of the dorsal fin there is a small, red-orange-coloured membrane. Yellowish thin vertical striations are visible on the abdominal area but quickly fade away ventrally. The head area is reddish-brown with vermiculation. The head area below the eye is light coloured, while the area above the eye has a darker red-brown colouration. A longitudinal brown stripe runs through the eye area.

The banding pattern of the *S. papilionaceus* (Fig. 1) specimens examined in this study was composed of two wide dorso-ventral brown bands, at times each appearing as composed of multiple fused bands. The first anterior brown band, occurring between the head area and the membrane of the eighth hard spine of the dorsal fin, fades ventrally at the abdomen and was found to be longer than the bands noted in *S. scriba.* This brown band is followed by a lighter cream-coloured band originating between the last few hard spines and the first few soft spines of the dorsal fin. This light-coloured band ends with a blue-violet patch at the abdomen. The latter was not present in all specimens we examined, and when present, it was less conspicuous than that found on *S. scriba.* This light-coloured band is followed by another dorso-ventral, wide, brown band originating from the soft spines of the dorsal fin and which appears as being composed of multiple fused bands. A brown banding pattern is also visible on the tail peduncle. The pectoral fins, pelvic fins, and tail, are brownish-orange. The anal fin and the membrane of the soft-rays of the dorsal fin have red-orange spots that are more pronounced than those of *S. scriba* and are separated by bluish lines. The tip of each hard spine of the dorsal fin has a small, red-orange-coloured membrane. The head area of *S. papilionaceus* is brownish as opposed to the red-brown of *S. scriba.* In *S. papilionaceus*, the head area below the eye is lighter in colour than the dorsal area of the head. The vermiculation on the head is more pronounced in *S. papilionaceus* than in *S. scriba*, and as for the former species, the lighter patterns contrast more against the darker brown colouration. In *S. papilionaceus*, the brown longitudinal stripe that runs through the eye area is not as conspicuous as that of *S. scriba.*

Most of the vivid colourations noted on both *S. scriba* and *S. papilionaceus* are mostly visible on live specimens, and some of the details are lost once the individuals die; however, the main brown bands tend to remain visible for a longer time and remain persistent in some old museum preserved specimens (Suppl. material 1: Fig. S2).

**Table 3.** External measures of *Serranus papilionaceus* and *Serranus scriba* expressed as a percentage of the total length.

Meristic measures	* Serranus papilionaceus*			* Serranus scriba*			* Serranus scriba*		
	Canary Islands			Malta			Turkey		
	current study (*n* = 5)			current study (*n* = 15)			Zorica et al. 2010 (*n* = 253)		
	range	mean	SD	range	mean	SD	range	mean	SD
Total length (mm)	187–245	216.6	±26.3	101–205	131.8	±30.9	71–200	110	±17.0
Standard length	82.9–85.7	84.5	±1.2	82.6–88.3	85.3	±1.6	74.7–94.3	84.3	±1.8
Head length	28.9–33.9	31.6	±2.0	31.8–35.6	33.8	±1.1	16.8–40.0	33.5	±1.6
Preocular head length	8.9–10.0	9.4	±0.4	8.0–10.2	9.4	±0.6			
Eye diameter	4.8–5.3	5.1	±0.3	4.9–6.8	6.2	±0.6			
Postocular head length	14.3–17.6	16.0	±1.5	15.8–18.8	17.1	±0.8			
Predorsal distance	28.6–32.9	30.7	±1.6	31.2–35.9	33.7	±1.3	28.9–43.5	33.6	±1.9
Preanal distance	52.4–56.7	55.2	±1.6	53.0–59.2	56.4	±1.9	48.3–64.8	55.3	±1.9
Maximum body height	24.2–25.8	25.1	±0.6	22.9–28.7	25.5	±1.5	16.8–47.1	27.9	±2.6
Minimum body height	10.7–11.7	11.3	±0.4	9.0–10.2	9.7	±0.3			
Length of dorsal fin	45.3–47.6	46.3	±1.1	39.8–44.1	41.4	±1.4	31.0–47.4	40.9	±1.4
Length of anal fin	12.7–15.3	14.0	±1.1	12.0–14.2	12.9	±0.7	7.5–15.5	12.2	±1.3
Length of pectoral fin	18.4–23.0	21.1	±1.8	21.1–23.8	22.0	±0.8	17.4–27.8	22.5	±1.4
Length of ventral fin	17.6–21.6	19.3	±1.6	15.6–17.4	16.6	±0.5	10.3–21.0	17.6	±1.4
Length of caudal fin	16.3–18.6	17.0	±0.9	11.7–17.4	14.7	±1.6	5.7–18.4	15.7	±1.4

### Genetic investigations

#### Genetic divergence

**Figure 3.**
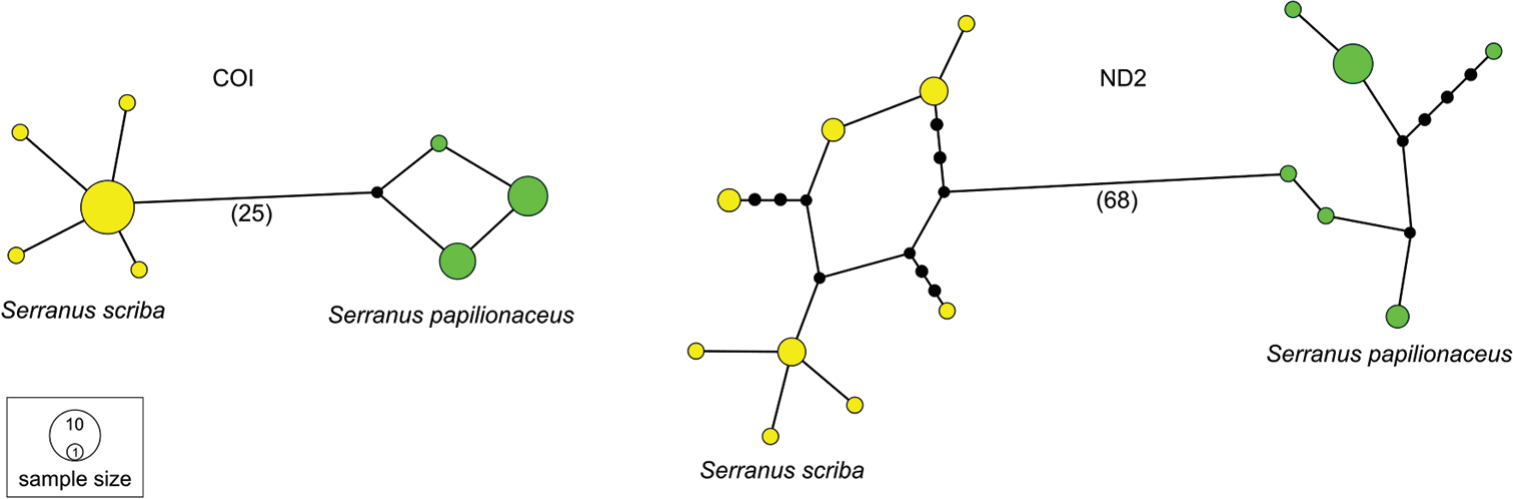
Two parsimony haplotype networks representing the genetic relationship between *Serranus papilionaceus* (Canary Islands) and *Serranus scriba* (Malta) for the COI and ND2 data (The haplotype frequencies are proportional with the area of the circle. The numbers in the brackets represent the number of interspecific substitutions, while the black circles represent inferred putative haplotypes within species that were not observed during this study).

**Figure 4.**
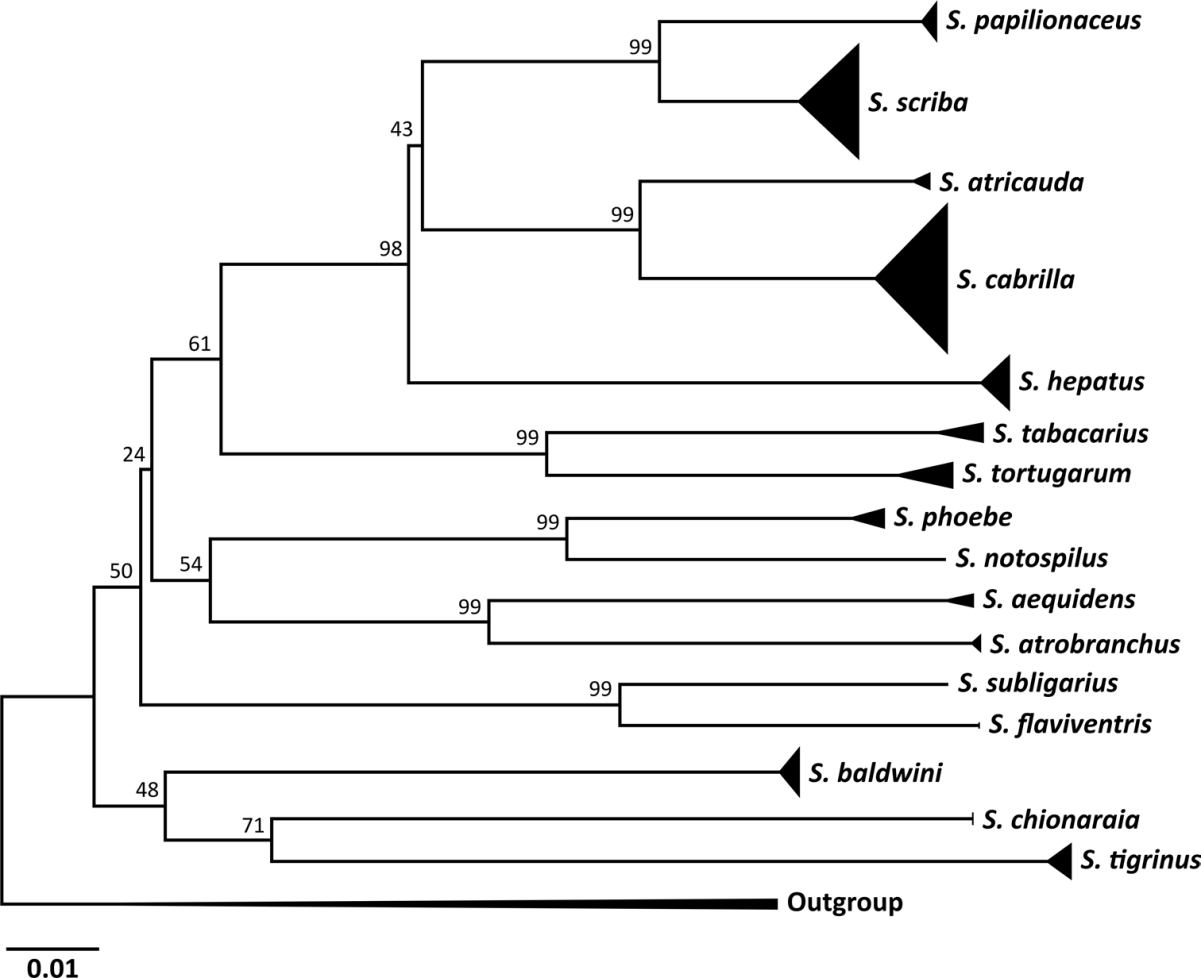
A neighbour-joining phylogenetic tree using p-distance. 186 COI sequences (578 bp each) representing 16 different *Serranus* species were utilized (Suppl. material 1: Table S3). The numbers near nodes represent the bootstrap values.

The COI and ND2 data produced three and six haplotypes for the specimens collected from the Canary Islands (*n* = 12), while five and nine haplotypes were identified amongst the specimens collected from Malta (*n* = 15) for each respective gene (Table 4; Fig. 3). The intraspecific divergence for COI of the analysed specimens from Malta was 0.09%, while on incorporating similar publicly available data for *S. scriba* from other areas of the Mediterranean (*n* = 41; Suppl. material 1: Table S3), the intraspecific divergence increased to 1.12%, with all sequences clustering together on the same branch of the phylogenetic tree (Fig. 4). For the specimens collected from the Canary Islands, the intraspecific divergence on COI was 0.11%. The genetic divergence between the two collections was found to be 4.31% (Table 5), that is four times larger than the intraspecific divergence, exceeding the species boundary delimitation of fish species (Ward et al. 2005). This led to the confirmation that specimens belonged to two different species. This large interspecific genetic difference was also noted on the ND2 gene where the intraspecific divergence was 0.45% and 0.31% for *S. scriba* and *S. papilionaceus*, respectively, while the interspecific divergence was 8.68% (Table 5), again surpassing the intraspecific divergence differences noted on the ND2 gene in other fish species by Naylor et al. (2012) and Vella et al. (2017). The haplotype networks of this mtDNA data show that the two species form two distinct clades, with no overlapping haplotypes (Fig. 3).

These results were further corroborated by both nuclear markers, where species specific SNPs were noted (Table 6). This data therefore confirms that genetically the specimens collected from the Canary Islands belong to a different species from those collected from Malta.

**Table 4.** The number of haplotypes noted (*hap.*), the haplotype diversity (*h*) and the nucleotide diversity (*π*) at each gene for *Serranus papilionaceus* and *Serranus scriba*.

mtDNA gene	* Serranus papilionaceus*			* Serranus scriba*		
	*hap*	*h*	*π*	*hap*	*h*	*π*
**COI** (621 bp)	3	0.621 ±0.087	0.0011 ±0.0010	5	0.476 ±0.155	0.0009 ±0.0009
**ND2** (846 bp)	6	0.758 ±0.122	0.0031 ±0.0020	9	0.924 ±0.044	0.0045 ±0.0027
**concatenated** (1467 bp)	7	0.833 ±0.100	0.0023 ±0.0014	11	0.952 ±0.040	0.0029 ±0.0017

**Table 5.** Intraspecific and intraspecific average number of pairwise differences (percentage differences) for the mtDNA loci of *Serranus papilionaceus* and *Serranus scriba*.

mtDNA gene	Intraspecific divergence		Interspecific divergence
	* Serranus papilionaceus*	* Serranus scriba*	
**COI** (621 bp)	0.70 (0.11%)	0.53 (0.09%)	26.77 (4.31%)
**ND2** (846 bp)	2.62 (0.31%)	3.79 (0.45%)	73.43 (8.68%)
**concatenated** (1467 bp)	3.32 (0.23)	4.33 (0.30%)	100.20 (6.83%)

**Table 6.** SNPs noted for the nDNA loci of *Serranus papilionaceus* and *Serranus scriba* (^a^ species-specific differences).

nDNA gene	Rhod							PTR	
Position	30	204	225^a^	232	273	628^a^	690^a^	160^a^	205^a^
* Serranus papilionaceus*	C (*n* = 11)	C (*n* = 15)	G (*n* = 12)	T (*n* = 9)	C (*n* = 9)	A (*n* = 15)	C (*n* = 15)	T (*n* = 12)	G (*n* = 12)
	C/T (*n* = 1)			C/T (*n* = 3)	C/T (*n* = 3)				
* Serranus scriba*	C (*n* = 15)	C (*n* = 14)	T (*n* = 15)	C (*n* = 15)	C (*n* = 15)	G (*n* = 15)	T (*n* = 15)	C (*n* = 15)	A (*n* = 15)
		C/T (*n* = 1)							

#### Phylogeny

All the species of *Serranus* found in the Mediterranean and the Canary Islands (*S. atricauda*, *S. cabrilla*, *S. hepatus*, *S. papilionaceus*, and *S. scriba*) share the same ancestral branch of the phylogenetic tree (Fig. 4), with each species forming its own distinct clade. This phylogenetic tree placed *S. scriba* and *S. papilionaceus* as sister species to each other.

### Vernacular name

We suggest Butterfly-winged Comber (Le Serran à Ailes de Papillon) as a common name of the species referring to its scientific name and the first description by Valenciennes (1832).

### Distribution

Specimens of *S. papilionaceus* used in this work were collected from the coastal waters of the Canary Islands (Eastern Atlantic Ocean), the same archipelago where the species was recorded nearly two centuries ago by Barker-Webb and Berthelot (1836). The original description of Valenciennes (1832) also places this species at the island of Gorée off the coast of Senegal. Museum specimen MNHN-IC-1896-0389 reconfirms this species at Senegal, while specimens collected from Mauritania (MNHN-IC-1999-1053) indicate that this species occurs also along the coast of this country (Fig. 2). The species might be more widely distributed along the western African coast and, therefore, more studies are required to better evaluate its distribution.

## Discussion

Mitochondrial DNA data show that the genetic distance between the Mediterranean specimens of *S. scriba* and the ones collected from waters of the Canary Islands exceeded the intraspecific genetic distance (COI: 4.31%; ND2: 8.68%; Figs 3, 4) for both genes (Ward et al. 2005; Naylor et al. 2012; Vella et al. 2017), confirming that the specimens from these two different sampling sites belong to two different species. The morphological colour and banding pattern difference between these two species support the genetic distinction found.

*Serranus scriba* was first described by Linnaeus (1758: 292) as *Perca scriba*. This description is fairly brief and lacks the locality; however, specimen NRM 442 from the Swedish Museum of Natural History (Suppl. material 1: Fig. S1a) is believed to be the holotype for *P. scriba* (Fernholm and Wheller 1983; Parenti and Randall 2020). In *Museum Adolphi Friderici* volume 2, Linnaeus (1764: 86) updated the description of *Perca scriba* and included some more details on the colouration of the species, namely reticulations and undulations over the head and brownish transverse bands, relating it to the Mediterranean *S. scriba.* However, in this account Linnaeus (1764: 86) indicated America as its area of distribution (Fernholm and Wheller 1983). Cuvier and Valenciennes (1828) made a detailed description of *S. scriba*, providing an illustration of the species (plate 28; Suppl. material 1: Fig. S1b), as well as indicating its distribution as including the coast of Provence (France), Malta, Naples (Italy), and Alexandria (Egypt). This description matches the two museum specimens MNHN-IC-0000-7129 (Suppl. material 1: Fig. S2b) and the specimens collected from Malta for the current study (Fig. 1). We therefore keep the widely accepted *S. scriba* nomenclature here in reference to the Mediterranean species sampled and genetically investigated in this study.

After reviewing the possible junior synonyms for *S. scriba* (Fernholm and Wheller 1983; Smith-Vaniz 2015; Horton et al. 2020; Parenti and Randall 2020), it was noted that our specimens from the Canary Islands matched the description of *S. papilionaceus* (Valenciennes, in Cuvier and Valenciennes 1832: 471). *Serranus papilionaceus* was first collected from Gorée Island, Senegal, by M. Rang and described by Valenciennes (1832: 471), and it is represented by two extant syntypes in the Muséum National d’Histoire Naturelle (MNHN-IC-0000-7279; Suppl. material 1: Fig. S2a). Our findings corroborate the second account for this species as described by Barker-Webb and Berthelot (1836), who indicated that *S. papilionaceus* occurs in the Canary Islands. In the current study, we also reviewed the description of *H. marocannus* Bloch & Schneider, 1801, as Peters (1865) suggested that this is synonymous to *S. papilionaceus* Valenciennes, 1832. However, given that the original description (Bloch and Schneider 1801: 320) and the quality of the type specimen (ZMB5531) lack the morphological details required to synonymise the two, we consider *H. marocannus* to be a junior synonym of *S. scriba* as noted by Cuvier and Valenciennes (1828), Gunther (1859), and Parenti and Randall (2020).

Historic records show that *S. papilionaceus* was accepted as a valid species name for a couple of decades (Barker-Webb and Berthelot 1836; Gunther 1859). However, by 1890, studies on specimens from the Canary Islands were considered as the variant S. scriba var. papilionaceus (Dooley et al. 1985). As a result, the Butterfly-winged Comber had lost its status as a distinct species. The similarity between the two species was reported since the first description of *S. papilionaceus*, with Barker-Webb and Berthelot (1836) stating that the two species, considered here, were very similar and could be easily misidentified. Nonetheless, the banding pattern, especially of live specimens is the most distinctive morphological feature to differentiate between the two species. Additionally, the results of our genetic analyses confirm the genetic divergence between these two species.

The resurrection of *S. papilionaceus* adds another species to the genus *Serranus*, genetically sister to *S. scriba* (Fig. 4). This calls for new studies that look into the geographical distribution of both *S. papilionaceus* and *S. scriba.* Online searches for *S. scriba* indicate that the morphological details belonging to *S. papilionaceus* are present in photos that have originated from different coastal regions along the North-Eastern Atlantic Ocean, and we cannot exclude the possibility of potential range overlap of the two species in certain geographical areas.

Though *Serranus* species have been listed as Least Concern (Smith-Vaniz 2015; IUCN 2020), the conservation status of the now recognised distinct species, *S. papilionaceus*, needs to be evaluated. This is especially so when one considers that the Canary Islands had already listed their *Serranus* species as threatened some time ago (Bonnet and Rodriguez 1992; Tuset et al. 2005) and fishing pressures have not decreased.

## Conclusion

Genetic diversity investigation results and different body colour patterns observed have led us to resurrect *S. papilionaceus*Valenciennes 1832 as a species distinct from *S. scriba* (Linnaeus, 1758). Phylogenetic analysis confirmed that these two species are sister species within the genus *Serranus* and that their interspecific genetic divergence is four times larger than the usual intraspecific divergence in fish species. The resurrection of *S. papilionaceus* also emphasizes the required new research to understand the distinct distributions, threats, and conservation management of these species.

## Supplementary materials

Supplementary material 1 Tables S1–S3, Figures S1, S2 Data type tables and images https://binary.pensoft.net/file/509278This dataset is made available under the Open Database License (http://opendatacommons.org/licenses/odbl/1.0/). The Open Database License (ODbL) is a license agreement intended to allow users to freely share, modify, and use this Dataset while maintaining this same freedom for others, provided that the original source and author(s) are credited.Adriana Vella, Noel Vella, Carolina Acosta-Díaz
