# Supplementary material for: Resurrection of the Butterfly-winged Comber, Serranus papilionaceus Valenciennes, 1832 (Teleostei, Serranidae) and its phylogenetic position within genus Serranus
Source: Zookeys. 2021 Feb 12;1017:111–26. doi: 10.3897/zookeys.1017.60637 (PMC7895806; doi:10.3897/zookeys.1017.60637)
Supplement: Supplementary material 1 — Tables S1–S3, Figures S1, S2 [file zookeys-1017-111-s001.pdf]

Supplementary Material:

Resurrection of the Butterfly-winged Comber, *Serranus papilionaceus* Valenciennes, 1832 (Teleostei: Serranidae) and its phylogenetic position within genus *Serranus*.

Adriana Vella, Noel Vella and Carolina Acosta-Díaz

Corresponding author: adriana.vella@um.edu.mt

Table S1. Details of the sampling locations of *Serranus papilionaceus* and *Serranus scriba* specimens that were analysed in the current study ([a] specimens utilized for measures and meristic counts).

Species / Specimen code	Date of collection	FAO Area / Location	GPS
S. papilionaceus		**GSA 34**	
Spap001	19/04/2017	Santa Cruz de Tenerife, Canary Islands	28.5111, -16.1722
Spap002	19/04/2017	Santa Cruz de Tenerife, Canary Islands	28.5111, -16.1722
Spap003	20/04/2017	Santa Cruz de Tenerife, Canary Islands	28.5097, -16.1707
Spap004	28/04/2017	Santa Cruz de Tenerife, Canary Islands	28.5108, -16.1738
Spap005	28/04/2017	Santa Cruz de Tenerife, Canary Islands	28.5108, -16.1729
Spap006	28/04/2017	Santa Cruz de Tenerife, Canary Islands	28.5108, -16.1729
Spap007	28/04/2017	Santa Cruz de Tenerife, Canary Islands	28.5108, -16.1729
Spap008[a]	12/05/2017	Santa Cruz de Tenerife, Canary Islands	28.5083, -16.1763
Spap009[a]	15/06/2017	Santa Cruz de Tenerife, Canary Islands	28.5083, -16.1763
Spap010[a]	14/06/2017	Garachico, Tenerife, Canary Islands	28.3799, -16.7600
Spap011[a]	14/06/2017	Garachico, Tenerife, Canary Islands	28.3799, -16.7600
Spap012[a]	14/06/2017	Garachico, Tenerife, Canary Islands	28.3799, -16.7600
S. scriba		**GSA 15**	
Sscr160117137[a]	17/01/2016	Grand Harbour, Valletta, Malta	35.8936, 14.5123
Sscr160117141[a]	17/01/2016	Grand Harbour, Valletta, Malta	35.8936, 14.5123
Sscr160117145[a]	17/01/2016	Grand Harbour, Valletta, Malta	35.8936, 14.5123
Sscr160130016[a]	30/01/2016	Cirkewwa, Mellieha, Malta	35.9896, 14.3287
Sscr160131002[a]	31/01/2016	Marsascala, Malta	35.8679, 14.5747
Sscr160214086[a]	14/02/2016	Xghajra, Malta	35.8815, 14.5511
Sscr160214087[a]	14/02/2016	Xghajra, Malta	35.8815, 14.5511
Sscr160214090[a]	14/02/2016	Xghajra, Malta	35.8815, 14.5511
Sscr160417002[a]	17/04/2016	Marsamxett Harbour, Valletta, Malta	35.9021, 14.5121
Sscr160501178[a]	01/05/2016	Armier, Mellieha, Malta	35.9920, 14.3606
Sscr160515174[a]	15/05/2016	Mellieha, Malta	35.9691, 14.3714
Sscr160605005[a]	05/06/2016	Marsamxett Harbour, Valletta, Malta	35.9022, 14.5124
Sscr160620055[a]	20/06/2016	Marsamxett Harbour, Valletta, Malta	35.9026, 14.5139
Sscr160626035[a]	26/06/2016	Mtahleb, Rabat, Malta	35.8771, 14.3395
Sscr160626036[a]	26/06/2016	Mtahleb, Rabat, Malta	35.8771, 14.3395

Table S2. Details of the *Serranus papilionaceus* and *Serranus scriba* GenBank accession numbers related to each specimen analysed in the current study.

Species<br>Specimen code	COI	ND2	Rhod	PTR
S. papilionaceus				
Spap001	MW439283	MW447431	MW447458	MW447485
Spap002	MW439284	MW447432	MW447459	MW447486
Spap003	MW439285	MW447433	MW447460	MW447487
Spap004	MW439286	MW447434	MW447461	MW447488
Spap005	MW439287	MW447435	MW447462	MW447489
Spap006	MW439288	MW447436	MW447463	MW447490
Spap007	MW439289	MW447437	MW447464	MW447491
Spap008	MW439290	MW447438	MW447465	MW447492
Spap009	MW439291	MW447439	MW447466	MW447493
Spap010	MW439292	MW447440	MW447467	MW447494
Spap011	MW439293	MW447441	MW447468	MW447495
Spap012	MW439294	MW447442	MW447469	MW447496
S. scriba				
Sscr160117137	MW439295	MW447416	MW447443	MW447470
Sscr160117141	MW439296	MW447417	MW447444	MW447471
Sscr160117145	MW439297	MW447418	MW447445	MW447472
Sscr160130016	MW439298	MW447419	MW447446	MW447473
Sscr160131002	MW439299	MW447420	MW447447	MW447474
Sscr160214086	MW439300	MW447421	MW447448	MW447475
Sscr160214087	MW439301	MW447422	MW447449	MW447476
Sscr160214090	MW439302	MW447423	MW447450	MW447477
Sscr160417002	MW439303	MW447424	MW447451	MW447478
Sscr160501178	MW439304	MW447425	MW447452	MW447479
Sscr160515174	MW439305	MW447426	MW447453	MW447480
Sscr160605005	MW439306	MW447427	MW447454	MW447481
Sscr160620055	MW439307	MW447428	MW447455	MW447482
Sscr160626035	MW439308	MW447429	MW447456	MW447483
Sscr160626036	MW439309	MW447430	MW447457	MW447484

Table S3. List of species and GenBank Accession numbers for the COI sequences used in the construction of the phylogenetic tree.

Species	BOLD BIN ID	Reference
**GenBank ID / BOLD ID**	**Sampling location**	
S. scriba	**BOLD:AAN9242**	
KC501473	Turkey	Keskin and Atar, 2013
KC501474	Turkey	Keskin and Atar, 2013
KC501475	Turkey	Keskin and Atar, 2013
KC501476	Turkey	Keskin and Atar, 2013
KC501477	Turkey	Keskin and Atar, 2013
KC501478	Turkey	Keskin and Atar, 2013
KC501479	Turkey	Keskin and Atar, 2013
KC501480	Turkey	Keskin and Atar, 2013
KC501481	Turkey	Keskin and Atar, 2013
KC501482	Turkey	Keskin and Atar, 2013
KC501483	Turkey	Keskin and Atar, 2013
KC501484	Turkey	Keskin and Atar, 2013
KC501485	Turkey	Keskin and Atar, 2013
KC501486	Turkey	Keskin and Atar, 2013
KC501487	Turkey	Keskin and Atar, 2013
KC501488	Turkey	Keskin and Atar, 2013
KC501489	Turkey	Keskin and Atar, 2013
KC501490	Turkey	Keskin and Atar, 2013
KC501491	Turkey	Keskin and Atar, 2013
KC501492	Turkey	Keskin and Atar, 2013
KJ709632	Sicily, Italy	Landi et al. 2014
KX925354	Malta	Vella and Vella, 2016
KX925355	Malta	Vella and Vella, 2016
KX925356	Malta	Vella and Vella, 2016
KX925357	Malta	Vella and Vella, 2016
MW439295	Malta	current study
MW439296	Malta	current study
MW439297	Malta	current study
MW439298	Malta	current study
MW439299	Malta	current study
MW439300	Malta	current study
MW439301	Malta	current study
MW439302	Malta	current study
MW439303	Malta	current study
MW439304	Malta	current study
MW439305	Malta	current study
MW439306	Malta	current study
MW439307	Malta	current study
MW439308	Malta	current study
MW439309	Malta	current study
S. papilionaceus		
MW439283	Canary Islands	current study
MW439284	Canary Islands	current study
MW439285	Canary Islands	current study
MW439286	Canary Islands	current study
MW439287	Canary Islands	current study
MW439288	Canary Islands	current study
MW439289	Canary Islands	current study
MW439290	Canary Islands	current study
MW439291	Canary Islands	current study
MW439292	Canary Islands	current study
MW439293	Canary Islands	current study
MW439294	Canary Islands	current study
S. atricauda	**BOLD:ACB3209**	
KX925333	Canary Islands	Vella and Vella 2016
KX925334	Canary Islands	Vella and Vella 2016
KX925335	Canary Islands	Vella and Vella 2016
KX925336	Canary Islands	Vella and Vella 2016
KX925337	Canary Islands	Vella and Vella 2016
S. cabrilla	**BOLD:AAD1027**	
KC501453	Turkey	Keskin and Atar 2013
KC501454	Turkey	Keskin and Atar 2013
KC501455	Turkey	Keskin and Atar 2013

Species	BOLD BIN ID	Reference
**GenBank ID / BOLD ID**	**Sampling location**	
***S. cabrilla* (cont.)**		
KC501456	Turkey	Keskin & Atar 2013
KC501457	Turkey	Keskin & Atar 2013
KC501458	Turkey	Keskin & Atar 2013
KC501459	Turkey	Keskin & Atar 2013
KC501460	Turkey	Keskin & Atar 2013
KC501461	Turkey	Keskin & Atar 2013
KC501462	Turkey	Keskin & Atar 2013
KC501463	Turkey	Keskin and Atar 2013
KC501464	Turkey	Keskin and Atar 2013
KC501465	Turkey	Keskin and Atar 2013
KC501466	Turkey	Keskin and Atar 2013
KC501467	Turkey	Keskin and Atar 2013
KC501468	Turkey	Keskin and Atar 2013
KC501469	Turkey	Keskin and Atar 2013
KC501470	Turkey	Keskin and Atar 2013
KC501471	Turkey	Keskin and Atar 2013
KC501472	Turkey	Keskin and Atar 2013
KJ709623	Sicily, Italy	Landi et al. 2014
KJ709624	Sicily, Italy	Landi et al. 2014
KJ709625	Sicily, Italy	Landi et al. 2014
KJ709626	Sicily, Italy	Landi et al. 2014
KJ709627	Sicily, Italy	Landi et al. 2014
KJ709628	Sicily, Italy	Landi et al. 2014
KJ709630	Sicily, Italy	Landi et al. 2014
KJ709902	Malta	Landi et al. 2014
KJ709903	Malta	Landi et al. 2014
KJ709904	Malta	Landi et al. 2014
KJ709905	Malta	Landi et al. 2014
KJ709906	Malta	Landi et al. 2014
KJ709907	Malta	Landi et al. 2014
KJ709908	Malta	Landi et al. 2014
KJ768309	Portugal	Landi et al. 2014
KX925338	Malta	Vella and Vella, 2016
KC501465	Turkey	Keskin and Atar 2013
KX925339	Malta	Vella and Vella 2016
KX925340	Malta	Vella and Vella 2016
KX925341	Malta	Vella and Vella 2016
KX925342	Malta	Vella and Vella 2016
KX925343	Malta	Vella and Vella 2016
KX925344	Malta	Vella and Vella 2016
KX925345	Malta	Vella and Vella 2016
KX925346	Canary Islands	Vella and Vella 2016
KX925347	Canary Islands	Vella and Vella 2016
KX925348	Canary Islands	Vella and Vella 2016
KX925349	Canary Islands	Vella and Vella 2016
S. hepatus	**BOLD:AAC6488**	
JQ774733	Portugal	Costa et al. 2012
JQ774734	Portugal	Costa et al. 2012
JQ774735	Portugal	Costa et al. 2012
JQ774736	Portugal	Costa et al. 2012
JQ774737	Portugal	Costa et al. 2012
JQ774916	Portugal	Costa et al. 2012
JQ774917	Portugal	Costa et al. 2012
KJ709631	Sicily, Italy	Landi et al. 2014
KJ709909	Malta	Landi et al. 2014
KJ709910	Malta	Landi et al. 2014
KJ709911	Malta	Landi et al. 2014
KJ709912	Malta	Landi et al. 2014
KJ709913	Malta	Landi et al. 2014
KX925350	Malta	Vella and Vella 2016
KX925351	Malta	Vella and Vella 2016
KX925352	Malta	Vella and Vella 2016
KX925353	Malta	Vella and Vella 2016

Species	BOLD BIN ID	Reference
GenBank ID / BOLD ID	Sampling location	
S. tabacarius	**BOLD:AAC4779**	
FJ584104	Cuba	Steinke et al. 2009
JQ839887	Berry Islands	Weigt et al. 2012
JQ841001	Belize	Weigt et al. 2012
S. tortugarum	**BOLD:AAB3018**	
JQ841003	Belize	Weigt et al. 2012
JQ841004	Belize	Weigt et al. 2012
JQ841005	Belize	Weigt et al. 2012
JQ841006	Belize	Weigt et al. 2012
JQ841007	Belize	Weigt et al. 2012
JQ841008	Belize	Weigt et al. 2012
JQ841821	Belize	Weigt et al. 2012
JQ841822	Belize	Weigt et al. 2012
S. phoebe	**BOLD:AAU1502**	
KF461238	Alabama, USA	Handy et al. unpub
KT075298	North Carolina, USA	Trizna, unpub
KT075320	North Carolina, USA	Trizna, unpub
MH378558	USA	Redmond et al. unpub
MH378563	USA	Redmond et al. unpub
MH378573	USA	Redmond et al. unpub
S. notospilus	**BOLD:AAH9574**	
KF930433	Mid Atlantic bight, USA	Bentley and Wiley unpub
S. aequidens	**BOLD:AAZ5418**	
MF957035	Costa Rica (Pacific)	Robertson et al. 2017
MF957036	Panama (Pacific)	Robertson et al. 2017
MF957037	Costa Rica (Pacific)	Robertson et al.2017
MF957038	Costa Rica (Pacific)	Robertson et al. 2017
S. atrobranchus	**BOLD:AAU2343**	
MH378526	USA	Redmond et al. unpub
MH378556	USA	Redmond et al. unpub
MH378602	USA	Redmond et al. unpub
MH378606	USA	Redmond et al. unpub
MH378610	USA	Redmond et al. unpub
S. subligarius	**BOLD:ACC6821**	
KF461239	Alabama, USA	Handy et al. unpub
S. flaviventris	**BOLD:AAE1690**	
JQ840701	Belize	Weigt et al. 2012
JQ840999	Belize	Weigt et al. 2012

Species	BOLD BIN ID	Reference
GenBank ID / BOLD ID	Sampling location	
S. baldwini	**BOLD:AAA6921**	
FJ584099	Cuba	Steinke et al. 2009
FJ584100	Cuba	Steinke et al. 2009
FJ584101	Cuba	Steinke et al. 2009
FJ584102	Cuba	Steinke et al. 2009
JQ840694	Belize	Weigt et al. 2012
JQ841380	Belize	Weigt et al. 2012
JQ841381	Belize	Weigt et al. 2012
JQ841382	Belize	Weigt et al. 2012
JQ841383	Belize	Weigt et al. 2012
JQ841384	Belize	Weigt et al. 2012
JQ841385	Belize	Weigt et al. 2012
JQ843053	Trinidad and Tobago	Weigt et al. 2012
JQ843054	Trinidad and Tobago	Weigt et al. 2012
JQ843055	Trinidad and Tobago	Weigt et al. 2012
JQ841387	Belize	Weigt et al. 2012
JQ841819	Belize	Weigt et al. 2012
JQ842315	Curacao	Weigt et al. 2012
S. chionaraia	**BOLD:AAC4820**	
LIDM897-07	Panama (Atlantic)	unpublished
MLIII396-08	Mexico (Atlantic)	unpublished
MLIII523-08	Belize	unpublished
MLIII565-08	Belize	unpublished
S. tigrinus	**BOLD:AAB5163**	
FJ584105	Cuba	Steinke et al. 2009
FJ584106	Cuba	Steinke et al. 2009
JQ839888	Berry Islands	Weigt et al. 2012
JQ839889	Berry Islands	Weigt et al. 2012
JQ841002	Belize	Weigt et al. 2012
JQ841389	Belize	Weigt et al. 2012
JQ841820	Belize	Weigt et al. 2012
JQ842316	Curacao	Weigt et al. 2012
JQ842317	Curacao	Weigt et al. 2012
JQ843057	Curacao	Weigt et al. 2012
**Outgroup**		
JX135579	*Hyporthodus octofasciatus*	Zhuang et al. 2013
KC593372	*Epinephelus trimaculatus*	Zhuang et al. 2013
KC593376	*Aethaloperca rogaa*	Zhuang et al. 2013

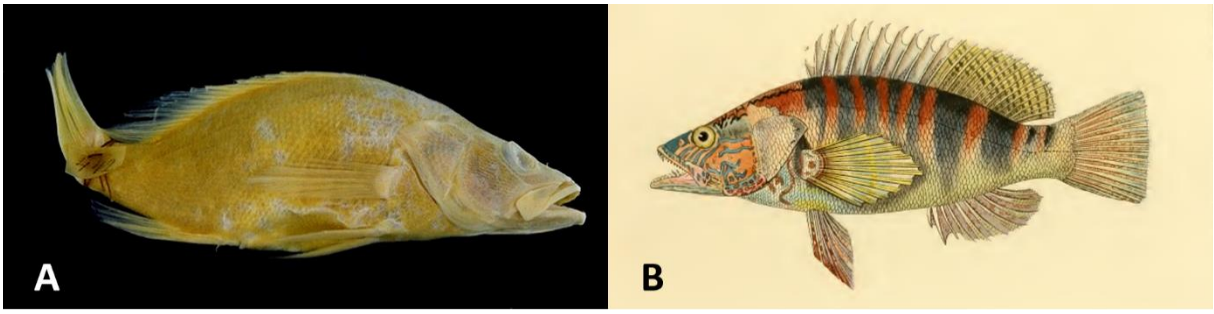

Figure S1. A NRM 442 is the oldest known type specimen for *Serranus scriba* [photo by Anders Silfvergrip, Swedish Museum of Natural History]. **B** An illustration representation of *Serranus scriba* (Plate 28) for the description of Cuvier and Valenciennes, 1828.

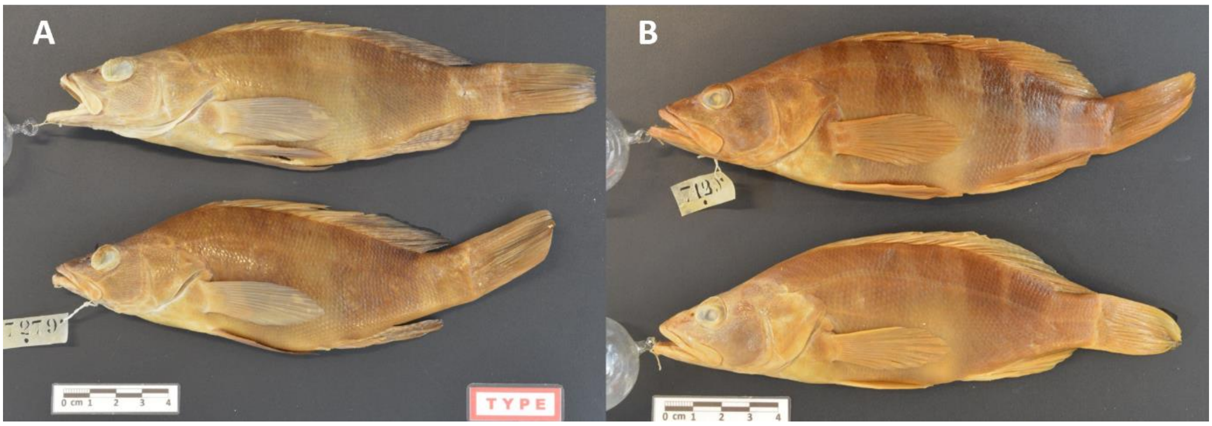

Figure S2. A MNHN-IC-0000-7279, two syntype specimens of *Serranus papilionaceus* from Gorée, Senegal (Atlantic Ocean) for the description of Valenciennes, 1832. **B** MNHN-IC-0000-7129, museum specimens of *Serranus scriba* from Algeria (Mediterranean Sea).
[© Muséum National d'Histoire Naturelle, Jonathan Pfliger]

References for Supplementary Material

Costa FO, Landi M, Martins, R, Costa MH, Costa ME, Carneiro M, Alves MJ, Steinke D, Carvalho GR (2012) A ranking system for reference libraries of DNA barcodes: application to marine fish species from Portugal. PloS one, 7(4), e35858.

Cuvier G, Valenciennes A (1828) Histoire naturelle des poissons. Tome Second. Chez F.G. Levrault. Paris, Strasbourg and Bruxelles, 490 pp.

Keskin E, Atar HH (2013) DNA barcoding commercially important fish species of Turkey. Molecular Ecology Resources, 13(5), 788-797.

Landi M, Dimech M, Arculeo M, Biondo G, Martins R, Carneiro M, Carvalho GR, Lo Brutto S, Costa FO (2014) DNA barcoding for species assignment: the case of Mediterranean marine fishes. PLoS One, 9, e106135.

Robertson DR, Angulo A, Baldwin CC, Pitassy D, Driskell A, Weigt L, Navarro IJF (2017) Deep-water bony fishes collected by the B/O Miguel Oliver on the shelf edge of Pacific Central America: an annotated, illustrated and DNA-barcoded checklist. Zootaxa, 4348, 1-125.

Steinke D, Zemlak TS, Hebert PD (2009) Barcoding nemo: DNA-based identifications for the ornamental fish trade. PloS one, 4(7), e6300.

Vella A, Vella N (2017) Genetic barcoding and preliminary phylogenetic analysis of Serranidae species from Maltese coastal waters, with a perspective on their Mediterranean phylogeography. Natural and Engineering Sciences, 1(3), 66-77.

Weigt LA, Baldwin CC, Driskell A, Smith DG, Ormos A, Reyier EA (2012) Using DNA Barcoding to Assess Caribbean Reef Fish Biodiversity: Expanding Taxonomic and Geographic Coverage. PLoS one, 7(7), e41059.

Zhuang X, Qu M, Zhang X, Ding S (2013) A comprehensive description and evolutionary analysis of 22 grouper (Perciformes, Epinephelidae) mitochondrial genomes with emphasis on two novel genome organizations. PloS one, 8(8), e73561.
